# Porous Nickel–Iron Oxide as a Highly Efficient Electrocatalyst for Oxygen Evolution Reaction

**DOI:** 10.1002/advs.201500199

**Published:** 2015-09-10

**Authors:** Jing Qi, Wei Zhang, Ruijuan Xiang, Kaiqiang Liu, Hong‐Yan Wang, Mingxing Chen, Yongzhen Han, Rui Cao

**Affiliations:** ^1^ School of Chemistry and Chemical EngineeringShaanxi Normal UniversityXi'an710119P.R. China; ^2^ Department of ChemistryRenmin University of ChinaBeijing100872P.R. China

**Keywords:** electrocatalysis, iron, nickel, oxygen evolution, water splitting

## Abstract

**A porous Ni–Fe oxide with improved crystallinity** has been prepared as a highly efficient electrocatalytic water oxidation catalyst. It has a small overpotential, a low Tafel slope, and an outstanding stability. The remarkably improved electrocatalytic performance is due to the porous structure, high extent homogeneous iron incorporation, ameliorative crystallinity, and the low mass transfer resistance.

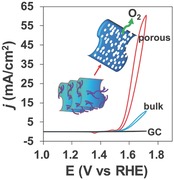

Water splitting for the generation of clean and sustainable energy resource represents one of the most promising processes toward environmental remediation.^1^ Oxygen evolution reaction (OER) is the bottleneck in water splitting as it is kinetically slow and requires high overpotential (*η*) to reach a substantial current density in water electrolysis.^2^ Broad attention has been therefore attracted to seek efficient OER electrocatalysts working at low overpotentials to reduce the energy input for water splitting.^3^ Regarding this, IrO_2_ and RuO_2_ were reported to have good electrocatalytic OER activity.[[qv: 3a]] However, the use of scarce noble metals limited their widespread applications. Toward identifying cheap and efficient electrocatalysts for OER, transition metal oxides,^4^ hydroxides,^5^ and oxyhydroxides^6^ based on earth abundant elements have been extensively examined. Among them, mixed‐metal materials containing iron group elements (Fe, Co, Ni) have been generally acknowledged as one of the most competent candidates for OER in aqueous media.[[qv: 3c]],[[qv: 4b‐d]],[[qv: 6c]],^7^

For the fabrication of iron group metal based materials as OER catalysts, one of the widely employed techniques is in situ electrodeposition method for the generation of amorphous films on the surface of electrodes. The structure and electrochemical activity of electrodeposited Ni, Co, Ni–Fe, Co–Fe, and Ni–Co films have been extensively studied.[[qv: 4a]],^6^, ^8^ Our recent work reported the first Fe‐based film prepared by a fast and simple cyclic voltammetry (CV) electrodeposition method as an OER electrocatalyst in neutral media with a high turnover frequency (TOF).[[qv: 5d]] Besides the electrodeposition route, photochemical deposition method for a soft loading of mixed Ni–Fe–Co film on electrode with controlled metal stoichiometry has also been developed.[[qv: 4b]] Alongside the deposition methods, mixed‐metal materials containing iron group elements have also been synthesized via a variety of techniques such as spin‐coating,^9^ pulsed‐laser ablation,^10^ aerosol spray,^11^ template synthesis,[[qv: 5c]],[[qv: 7a]] solid‐state reaction,^12^ as well as exfoliation of bulk materials^13^ toward improved electrochemical activity. Moreover, Ni–Fe layered double hydroxides (LDH) with the incorporation of carbon materials have been reported for significant enhancement of the OER activity.^14^

Typically, mixed Ni–Fe materials have shown excellent electrocatalytic activities in basic media.[[qv: 6a,c]],[[qv: 14d]] Ni is the active oxygen evolution center, and Fe incorporation will improve the conductivity of the material.[[qv: 4c,d]],[[qv: 6a]] It is universally acknowledged that high surface area will contribute to the activity improvement, whereas the poor crystallinity arisen from high surface area is adverse to the electrocatalytic stability. Thermal treatment of the materials for improved crystallinity can ameliorate their electrochemical stability.[[qv: 7a]],^12^ However, thermal treatment has been rarely applied to Ni–Fe electrocatalysts as it can largely reduce the surface area and thus the activity of the materials.[[qv: 4b]] Although considerable efforts have been devoted to fabricating electrode materials with optimized surface active sites via porous structure or 3D architecture,[[qv: 5b]],^7^,[[qv: 14a]],^15^ limited work has been documented on porous electrocatalysts with improved crystallinity for enhanced stability without the damage of their efficient OER performance. Thus, it is highly desirable to develop novel protocols for the synthesis of porous Ni–Fe materials with abundant surface active sites, and meanwhile, with improved crystallinity for ameliorative electrochemical stability.

Herein, we report a facile synthesis of porous Ni–Fe mixed oxides in the presence of organic surfactant Tween 85. By using Tween, which has a perfect boiling point for the synthesis of the catalyst in this work, the porous structure can be created at low temperature from evaporation of the mixed surfactant. While in traditional method, those carbon‐based surfactant templates are burnt out under high temperatures to get the porous structure. Heating treatment at elevated temperature not only requires large energy cost but also limits the application in thermal instable or unfavorable systems. Ni–Fe OER catalyst is a thermal unfavorable system. The creation of porous structure under mild temperature is not only suitable for the formation of active phase, but also preferable for the reservation of high surface area. The sample after thermal treatment under 200 °C has a porous structure and improved crystallinity compared to traditional Ni–Fe hydroxides synthesized under room temperature. This porous Ni–Fe catalyst outperformed its bulk material counterpart (Tween free sample) for OER. In CV studies, it can highly efficiently catalyze water oxidation in a 0.1 m KOH aqueous solution. Small overpotentials of 328 and 420 mV are required to reach OER current densities of 10 and 50 mA cm^−2^, respectively. In controlled potential electrolysis (CPE) at *η* = 387 mV, an extremely stable current density of ≈10.2 mA cm^−2^ maintained, giving an extraordinary TOF value of 432 h^−1^. Besides the outstanding performance of the material, our synthesis method is suitable for large‐scale production, and thus delivers a valuable contribution to electrocatalytic water splitting research.

The Ni–Fe composites were obtained through coprecipitation strategy in the presence of Tween 85, a polysorbate surfactant with a boiling point above 100 °C. The solids were collected and subjected to mild heat treatment under elevated temperatures for the removal of Tween to construct pores in the material. X‐ray diffraction (XRD) patterns for composites with different starting Ni–Fe ratios after calcination under 200 °C for 3 h (all the thermal treatment time is 3 h in this work), which is the thermal treatment condition toward the highest OER activity (see below), are shown in **Figure** **1**A. Samples are denoted as **Ni‐*X*‐*Y***, in which ***X*** stands for the starting Ni percentage and ***Y*** stands for the treatment temperature in degrees Celsius. The two samples with high nickel concentration tended to form β‐Ni(OH)_2_ (JCPDS 742075) phase showing selected better crystallinity from (100) and (110) reflections at 2*θ* degree of 33° and 59°, respectively. Increased Fe incorporation in **Ni‐90‐200** and **Ni‐85‐200** samples resulted in a dominant NiO (JCPDS 780643) phase with mixed weak signals from NiFe_2_O_4_ (JCPDS 742081) observed in the latter sample. The peaks around 62° and 75° in these two samples were assigned to NiO, as the characteristic XRD peak of NiFe_2_O_4_ at 30° was not observed. The iron incorporation in the composites can promote the transfer of hydroxides to oxides under thermal treatment, which is consistent to the X‐ray photoelectron spectroscopy (XPS) studies of Ni–Fe material by Smith et al.[[qv: 4b]] Samples with further increased iron content existed as NiFe_2_O_4_ with mixed NiO, and it was a mixture of NiFe_2_O_4_ and γ‐Fe_2_O_3_ (JCPDS 871166) for sample with even higher Fe content. For **Ni‐50‐200** sample, which contains actually over 94% of iron as suggested by XPS, the crystallinity is quite poor with a dominant γ‐Fe_2_O_3_ phase.

**Figure 1:**
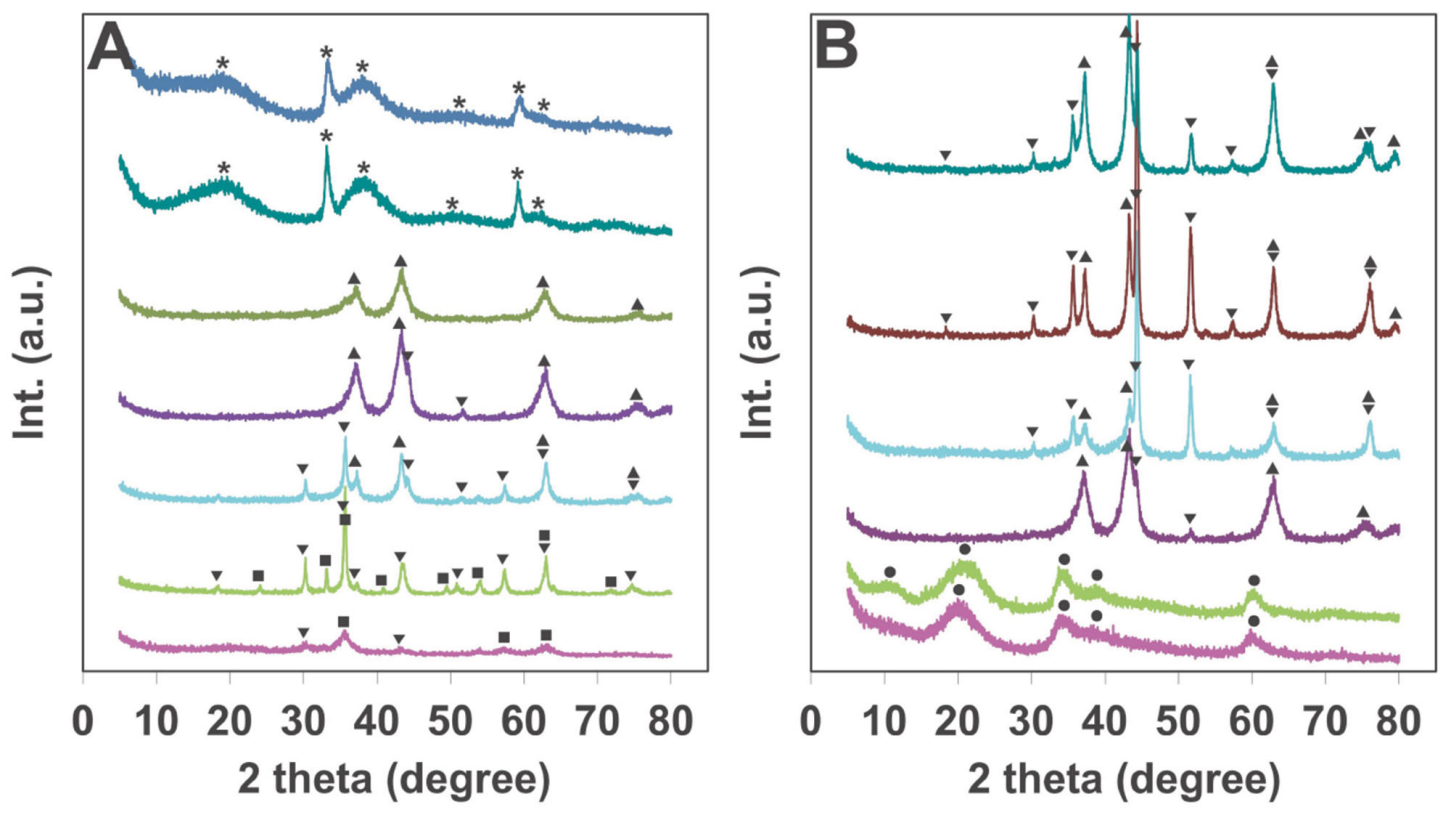
A) XRD patterns of the **Ni‐*X*‐200** samples (***X*** from bottom to top: 50, 70, 80, 85, 90, 98, 100); B) XRD patterns of the **Ni‐85‐*Y*** samples (***Y*** from bottom to top: rt, 100, 200, 300, 400, 500). ●: α‐Ni(OH)_2_, *: β‐Ni(OH)_2_, ▴: NiO, ▾: NiFe_2_O_4_, ◼: γ‐Fe_2_O_3_.

With a starting Ni–Fe ratio of 85:15, which is the tested Ni–Fe ratio toward the highest OER activity (see below), the synergic effects of heat treatment on crystal structure can be referred from the XRD patterns shown in Figure 1B. The freshly synthesized composite showed a structure that can be indexed to α‐Ni(OH)_2_ (JCPDS 380715). After heat treatment under 100 °C for 3 h, the α‐Ni(OH)_2_ phase maintained with an emerging peak around 11° from the (003) reflection perpendicular to the α‐Ni(OH)_2_ layers. This phenomenon resembles the structure conversion of electrodeposited Ni–Fe LDH with new XRD peak from (003) direction after aging in a hot solution for a period of time.[[qv: 6a]] Due to the high content of Tween in our samples as determined by element analysis (≈40 wt%), the Ni–Fe layers in the freshly prepared samples are probably wrapped by Tween molecules leading to the formation of Ni–Fe hydroxide nanosheets with limited layers. Upon heat treatment, Tween molecules were released. As a consequence, the Ni–Fe layers assembled together causing the XRD peak at 11° due to the diffraction from Ni–Fe hydroxide interlayers. The XRD result of the sample **Ni‐85‐200** without the addition of Tween shown in Figure S1 (Supporting Information) suggested a distinguishable crystalline phase of α‐Ni(OH)_2_, which is consistent with the above discussions on the formation of assembled layers in Tween wrapped samples. With elevated temperature treatment, the hydroxides start to form a dominant NiO (JCPDS 780643) structure under 200 °C and an NiO/NiFe_2_O_4_ (JCPDS 742081) composites starting from 300 °C.

As demonstrated in **Figure** **2**A, the release of Tween upon heat treatment can be verified from infrared spectra of the samples studied in Figure 1B. The red line sample was synthesized from the same conditions without Tween. The freshly prepared sample with Tween before heat treatment displays distinct peaks from C–H stretching vibrations of methylene and methyl groups (2860, 2940 cm^−1^), C=O/C–O stretching vibrations of ester/ether groups (1750, 1130 cm^−1^). Tween molecules were gradually removed from the sample after heat treatment, and only trace was left in the sample after 200 °C treatment. The O–H stretching vibration peaks (≈3480 cm^−1^) and scissoring vibration peaks (1700–1500 cm^−1^ from water, 1400–1300 cm^−1^ from structure hydroxyl groups) are obvious in all the samples, especially in samples under low‐temperature treatment. Thermal gravimetric analysis (TGA) and derivative thermogravimetric (DTG) analysis studies of the above mentioned sample are shown in Figure 2B. Upon elevated heat treatment, the mixture starts to lose weight from a sequence of absorbed water, structure water, Tween, and eventually undergoes a phase transfer from hydroxide to oxide.

**Figure 2:**
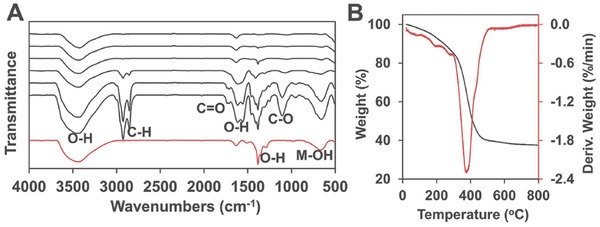
A) Infrared spectra of **Ni‐85‐*Y*** samples. (***Y*** of black lines from bottom to top: rt, 100, 200, 300, 400, 500; red line: **Ni‐85‐200** without Tween.) B) TGA and DTG analysis of the freshly prepared **Ni‐85‐rt** sample.

Upon the removal of Tween in the samples, porous structure was obtained and demonstrated by transmission electron microscopy (TEM) images (**Figure** **3**A–D), Brunauer–Emmett–Teller (BET) surface area, and pore size distribution measurements (Table S1, Supporting Information, and Figure 3E,F). After heat treatment, the bulk hydroxides (Figure 3A) turned into aggregates comprised of Ni–Fe domains (darker areas) and pores (lighter areas) as illustrated in Figure 3B,C. Higher temperature treatment converts the aggregates into more separated nanoparticles as shown in Figure 3D. Scanning electron microscope (SEM) and high‐resolution TEM (HRTEM) images of the **Ni‐85‐200** sample are provided in Figures S2 and S3 (Supporting Information). Well‐dispersed electrocatalysts on indium tin oxide (ITO) electrode was observed from the SEM image. In HRTEM, the lattice can be fairly indexed to the dominant reflection peaks of NiO. Elemental mapping of the material was demonstrated via energy dispersive X‐ray (EDX) spectroscopy, suggesting a homogeneous nickel/iron dispersion in the material as shown in Figures S4 and S5 (Supporting Information). BET surface areas and pore size distributions of the materials studied in TEM have been examined through N_2_ physical adsorption measurements. Figure 3E shows the pore size distribution of the aforementioned sample with a pore diameter around 3 nm. The pore size expanded with increasing temperature treatment. After calcination under 200 °C for 3 h, the pore volume of the sample with the addition of Tween during synthesis (red line, Figure 3E) is significantly higher than that from the sample without Tween (blue line, Figure 3E). The adsorption–desorption isotherm plot for the sample treated under 200 °C displays a type‐IV isotherm with a closure at *P*/*P*_0_ around 0.4, suggesting the presence of small mesopores in the material. Integrally, we are capable to illustrate the evolution procedure of the Ni–Fe material under heat treatment in **Figure** **4**. The freshly synthesized Tween wrapped Fe doped α‐Ni(OH)_2_ sheets assembled into layered Fe doped α‐Ni(OH)_2_ after heat treatment as the hydroxide sheets were less wrapped by Tween. Subsequently, it evolved into porous Fe‐doped NiO with the complete removal of Tween under higher temperature. With further elevated temperature treatment, the pore expanded and separated nanoparticles were formed eventually.

**Figure 3:**
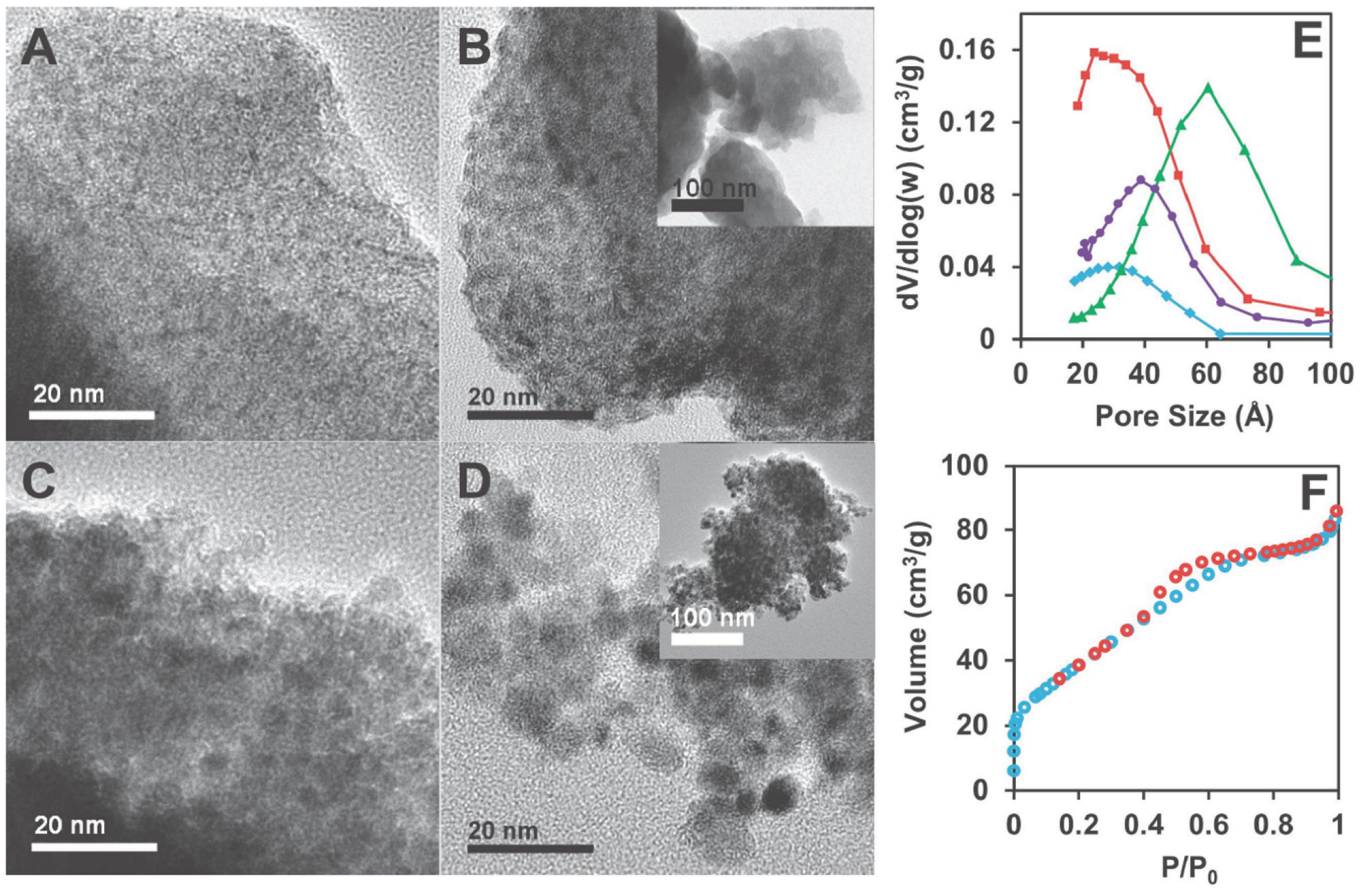
A–D) TEM images of the **Ni‐85‐*Y*** samples. ***Y*** values are A) rt; B) 200; C) 300; and D) 400. Insets are corresponding low‐magnification images. E) Pore size distribution of the **Ni‐85‐*Y*** samples. (***Y*** values: red, 200; purple, 300; green, 400; blue, 200, no Tween.) F) Adsorption–desorption isotherm for **Ni‐85‐200** sample.

**Figure 4:**
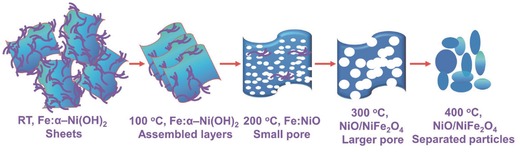
The evolution illustration of the as‐synthesized **Ni‐85‐rt** catalyst. The purple lines are Tween molecules.

The actual Ni–Fe composition information of the samples determined by XPS analysis is summarized in Table S1 (Supporting Information) with the foregoing discussed physical properties. The Ni/Fe 2p spectra for the **Ni‐*X*‐200** and **Ni‐85‐*Y*** samples are presented in Figure S6 (Supporting Information). The XPS results from the **Ni‐50‐*Y*** samples as displayed in gray line in Figure S6A,B (Supporting Information) indicate the very low nickel content, which is due to the much smaller solubility product from Fe(OH)_3_ (*K*_sp_ = 2.8 × 10^−39^) than Ni(OH)_2_ (*K*_sp_ = 5.5 × 10^−16^).^16^ Fe^III^ will be precipitated from basic solutions much easier than Ni^II^, leading to a low nickel content sample when base is not sufficient during synthesis. The Ni 2p and Fe 2p peaks for the **Ni‐85‐200** sample can be fairly resolved to support the XRD results of the composite (**Figure** **5**). A dominant Fe‐incorporated NiO phase and trace amount of NiFe_2_O_4_ was suggested from the XRD results. The Ni 2p_3/2_ peak centered at 855.5 eV is higher than that from pure NiO around 854 eV. The high extent doping of Fe^III^ with high electronegativity causes a higher valence state of Ni^II^.[[qv: 4b]],[[qv: 6a]] The small orange line peak with binding energy of 857.0 eV is assigned to the trace amount of isolated NiFe_2_O_4_, whose weak signals in XRD analysis can also be observed.^17^ The NiFe_2_O_4_ content is around 3.1 mol%. The Fe 2p_3/2_ peak is well fitted into the Fe^III^ signals at 710.2 and 715.6 eV based on the method developed for the spectrum of ferric with a nickel environment.^18^

**Figure 5:**
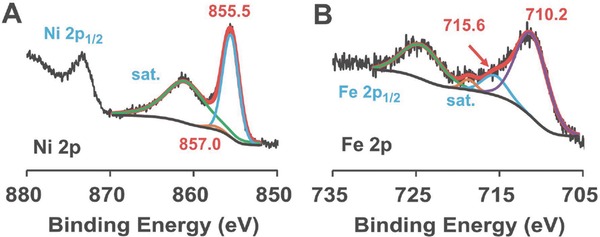
XPS spectra of A) Ni 2p and B) Fe 2p energy region from **Ni‐85‐200**.

The electrocatalytic performance of the porous Ni–Fe materials for water oxidation was tested. CVs of the **Ni‐*X*‐200** samples are shown in **Figure** **6**A. Pure nickel material has poor OER activity and displays distinct self‐redox peaks due to the oxidation of Ni^II^ to Ni^III^ at 1.45 V versus reversible hydrogen electrode (RHE, all potentials reported in this work are vs RHE).[[qv: 5b]],[[qv: 6a]] The addition of two percent of Fe during synthesis will significantly suppress the oxidation of Ni^II^. There is hardly notable Ni^II^ self‐redox peaks from samples with higher percentage of Fe incorporation. It is rationalized that Fe^III^ with high electronegativity can increase the valence state of surrounding nickel atoms, which become harder to be further oxidized. With the electron‐withdrawing effect on nickel, iron incorporation facilitates the partial charge transfer in the material for possible higher OER kinetics.[[qv: 6a,c]] The OER activity is remarkably improved as the starting Fe content increased to 15%. Further increase of the Fe content to 20% dramatically deteriorates the OER performance, and there is hardly notable water oxidation peak from the sample with 30% or higher starting Fe content.

**Figure 6:**
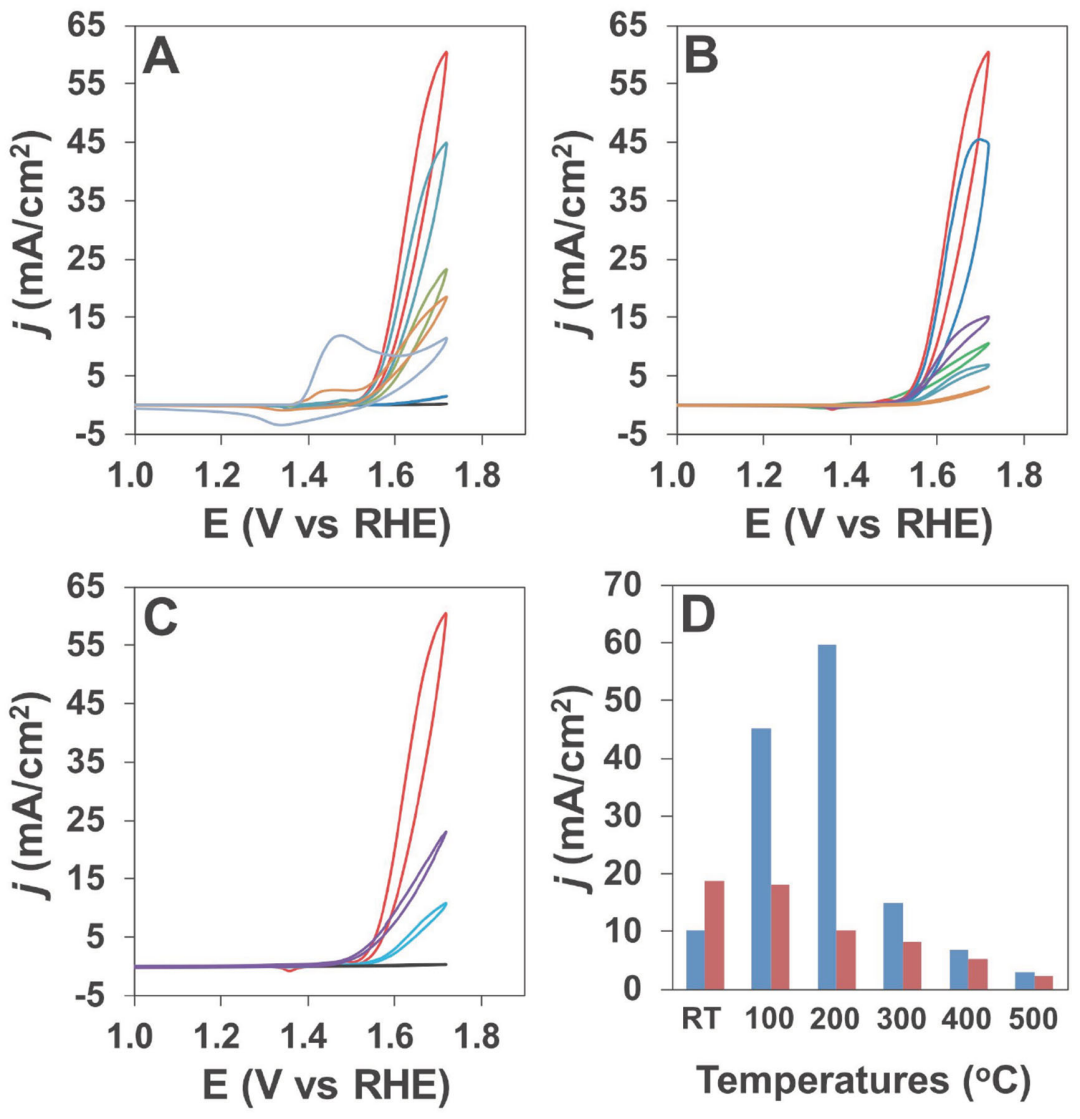
A) CVs of the **Ni‐*X*‐200** samples (***X*** from bottom to top: 50, 70, 100, 98, 80, 90, 85); B) CVs of the **Ni‐85‐*Y*** samples (***Y*** from bottom to top: 500, 400, rt, 300, 100, 200); and C) CVs of the best **Ni‐85‐200** sample (red) and the corresponding sample without Tween (blue) for comparison, the purple line is from Ir/C, the bottom line (black) is from blank GC electrode. D) Current densities under 1.72 V from the **Ni‐85‐*Y*** samples (blue: samples with Tween; red: samples without Tween).

Thermal treatment effects on the electrocatalytic activity have also been studied for the **Ni‐85‐*Y*** samples (Figure 6B). Thermal treatment of the freshly prepared sample at 100 °C for 3 h can improve its OER performance notably. For the **Ni‐85‐200** sample, the current can reach as high as 62 mA cm^−2^ at 1.72 V (*η* = 487 mV). Thermal treatment under further increased temperature will remarkably decrease the OER activity, probably due to the reduced surface area as well as structure change of the material. As proved by XRD analysis, the mixture was in the form of Fe‐doped NiO after 200 °C calcination. After higher temperature treatment, it turned into a mixture of NiO and NiFe_2_O_4_, which neither is reported to have excellent OER performance.[[qv: 3c]],[[qv: 4c]],^12^ As for **Ni‐85‐200** sample, the trace amount of NiFe_2_O_4_ in the sample is not believed to deteriorate the OER performance too much. Meanwhile, the Fe content is a significant determinant for the activity of Ni–Fe materials. Thus, the higher activity from the **Ni‐85‐200** is due to the saturated and optimally doped Fe content in the NiO phase. For comparison, Figure 6C shows the CV of the best porous **Ni‐85‐200** sample, the CV from the corresponding bulk sample synthesized without Tween, and the CV from blank glassy carbon (GC) electrode. The porous sample dominantly outperformed the bulk Ni–Fe hydroxide that is reported extensively as an efficient OER electrocatalyst. In addition, the benchmarked Ir/C (20 wt% of Ir) electrocatalyst was drop‐casted onto the GC electrode for comparison. Its CV study from the same condition showed a less active performance compared to the porous Ni–Fe oxides, although an earlier onset potential for OER was observed. The obtained porous Ni–Fe oxides can efficiently catalyze water oxidation with small overpotentials of 328 and 420 mV to reach current densities of 10 and 50 mA cm^−2^, respectively.

To better evaluate our electrocatalysts, a comparison of the current densities from the samples with/without the addition of Tween during synthesis is shown in Figure 6D. For the freshly prepared samples, the addition of Tween can suppress the OER activity, which is due to the blocking of surface active sites by large Tween molecules. With elevated heat treatment, the Tween molecules were removed steadily leaving a porous structure. As a consequence, it is reasonable to notice that the samples with Tween during synthesis greatly outperform the corresponding bulk material samples after heat treatment. It is also notable that high‐temperature calcination up to 400 °C made the structure difference less significant between plain and Tween added samples, and thus resulted in closer OER performances. The **Ni‐85‐100** sample with Tween has the same α‐Ni(OH)_2_ structure to the **Ni‐85‐200** sample without Tween. The higher performance from the former is probably due to the less assembled α‐Ni(OH)_2_ nanosheets as demonstrated from the XRD reflection from (003) facets around 11° as shown in Figure 1B and Figure S1 (Supporting Information). Tween wrapped sample can be converted from Ni(OH)_2_ to NiO under a relatively lower temperature, probably due to its poorer crystallinity. This feature helps to reserve the high surface area of the resultant active **Ni‐85‐200** sample. The detailed comparisons of the CV studies for samples with or without Tween are shown in Figure S7 (Supporting Information). The self‐redox peaks due to the oxidation of Ni^II^ to Ni^III^ were increasingly suppressed along with the increase of thermal treatment temperature. This is consistent with the increased valence state of Ni in the nickel ferrite after annealing under high temperature as studied by Solís et al.^19^ To this extent, thermal treatment causes a closer Ni–Fe interaction in the samples after calcination, which contributes to the improvement of electrocatalytic activity. Considering the reduced surface area after calcination, it is reasonable to observe the highest activity from the sample after thermal treatment under 200 °C.

As shown in **Figure** **7**A, CPE at 1.62 V was conducted in 0.1 m KOH aqueous solution using ITO electrodes casted with the porous **Ni‐85‐200** catalyst (red line) or without thermal treatment (purple line), and the corresponding catalyst without Tween (blue line). In CPE, a stable current density at ≈10.2 mA cm^−2^ maintained from the porous sample, indicating its outstanding electrocatalytic durability. Moreover, no activation process, which is widely reported for Ni–Fe based materials in water oxidation, is required to reach the high current density for this porous catalyst. These stability features are attributed to the improved crystallinity of our sample as a consequence of thermal treatment.[[qv: 7a]],^12^ The CPE results from the same sample without thermal treatment showed a much smaller current density and a rapid degradation of activity after 10 min of electrolysis. Before and after electrolysis, CVs were recorded and provided in Figure S8 (Supporting Information) for the samples with or without thermal treatment. The stable performance of the **Ni‐85‐200** is further expounded from the SEM and HRTEM images of the sample after electrolysis (Figure S9, Supporting Information), which suggested almost unchanged morphology and phase. Compared to the sample dried under room temperature, the porous sample showed much better stability due to its improved crystallinity after thermal treatment. With the Tween assisted synthesis, the mild heat treatment preserves the high surface area porous structure for efficient activity, and on the other hand, increases the crystallinity of the material for stability. For comparison, the sample without Tween during synthesis showed a much smaller current density of ≈2 mA cm^−2^ with a gradually increased current after the initial 1 h sluggish period. A movie of the vigorous evolution of oxygen bubbles during electrolysis under 1.62 V is provided (Movie S1, Supporting Information). The amount of evolved oxygen was measured using a calibrated Ocean Optics FOXY probe. During 6 h electrolysis, 55 C of charges passed with 141 μmol of O_2_ evolved, which gave a Faradaic yield of >98%. A high TOF value of 432 h^−1^ based on the nickel amount loaded was achieved, which is among those highest values reported for OER.[[qv: 4f]],[[qv: 5b]],[[qv: 6a,b]] It should be noted that the OER performance of metal oxides/hydroxides can be substantially improved in concentrated alkaline solution^11^,[[qv: 14d]],[[qv: 15d]] or from Au coated substrate electrodes.[[qv: 6a]],^20^ Thus, any comparison of the OER performance should be made based on the same experimental details.

**Figure 7:**
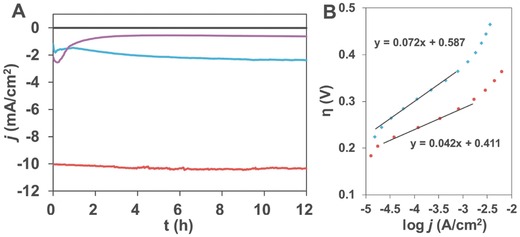
A) Current curves in CPE under 1.62 V from ITO electrodes casted with **Ni‐85‐200** catalysts with (red) or without (blue) Tween during synthesis. Purple line is the electrolysis result of the red line sample before thermal treatment. Black line is from blank ITO electrode. B) Tafel plots of **Ni‐85‐200** catalysts with Tween (red) and **Ni‐85‐250** catalysts without Tween (blue). Conditions: 0.1 m KOH aqueous solution, room temperature.

Figure 7B shows the Tafel plot of the porous sample and the bulk sample. A small Tafel slope of 42 mV decade^−1^ for the porous catalyst is achieved. Correspondingly, a higher value of 72 mV decade^−1^ is obtained for the bulk catalyst (**Ni‐85‐250** without Tween), which has the same NiO phase as **Ni‐85‐200** with Tween. The electrochemical impedance spectroscopy (EIS) was referred for more information of the electrocatalysts as shown in the Nyquist plots in **Figure** **8**. As confirmed from Figure 8A for the EIS of the porous sample under different potentials, the first semicircle in the high‐frequency region represents the charge transfer resistance from the bulk electrolyte to the catalyst surface, which is independent of the potential applied. The following second semicircle in the low‐frequency region represents the resistance from the mass transfer during electrocatalytic reactions, which is the reference of the redox reaction efficiency. The porous electrocatalyst has a much lower mass transfer resistance in OER as shown in Figure 8B, which is attributed to the porous structure. The ESI plots of the **Ni‐85‐*Y*** samples are provided in Figure S10 (Supporting Information) for reference. The lowest mass transfer resistance is from the **Ni‐85‐200** sample, which is the most active catalyst studied in this work.

**Figure 8:**
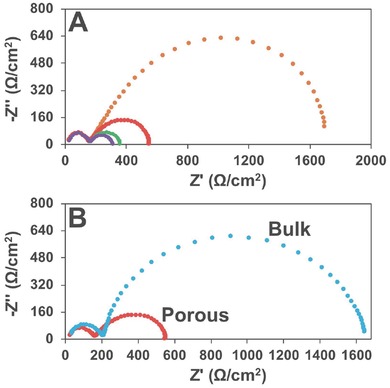
A) EIS Nyquist plots of **Ni‐85‐200** under different potentials (yellow: 1.52 V; red: 1.57 V; green: 1.62 V; purple: 1.67 V). B) EIS Nyquist plots of **Ni‐85‐200** (red) and corresponding bulk sample (blue) under 1.57 V.

In summary, a porous Ni–Fe oxide has been prepared with the assistance of Tween molecules. Upon mild thermal treatment, the polysorbate was removed gradually from the composite leaving the porous structure material with large surface area. Likewise, the crystallinity was improved and a homogeneous high extent Fe doping in NiO was achieved by heat treatment. The material is highly efficient for electrocatalytic water oxidation. From the porous sample, small overpotentials of 328 and 420 mV are required for OER to reach current densities of 10 and 50 mA cm^−2^, respectively. The current density of ≈10.2 mA cm^−2^ stays almost constant under 1.62 V electrolysis. The remarkably improved electrocatalytic performance compared to the bulk material is due to the porous structure, homogeneous Fe incorporation, ameliorative crystallinity, and the low mass transfer resistance.

## Experimental Section

*Synthesis of Electrocatalysts*: The materials were obtained through coprecipitation strategy with the presence of Tween 85, a polysorbate surfactant with a boiling point above 100 °C. The oily polysorbate of 10 mL was first dissolved in 50 mL of 1 m NaOH solution at 60 °C under vigorous stirring, and then a mixed aqueous solution containing Ni(NO_3_)_2_ and Fe(NO_3_)_3_ at different ratios (the total metal concentration is 2.5 m, volume is 20 mL) was added into the hot basic solution dropwise. The suspension was stirred under the given temperature for 4 h. The solids were collected and washed by centrifugation in a sequence of water (two times), acetone (one time), and water (two times) thoroughly. The obtained solids were dried in an oven at 60 °C and were subjected to further thermal treatment under different temperatures as required. The bulk samples for comparison were synthesized from the same procedure except the addition of Tween.

*Electrochemical Studies*: All electrochemical experiments were carried out using a CH Instruments (CHI 660E Electrochemical Analyzer) at 20 °C. CVs were obtained in 15 mL of 0.1 m KOH aqueous solution using a conventional three‐electrode configuration with a 0.07 cm^2^ GC electrode as the working electrode, saturated Ag/AgCl as the reference electrode, and Pt wire as the auxiliary electrode. The working electrode was prepared through a drop‐casting method. Typically, 2 mg of sample and 30 μL of Nafion solution (5 wt%, DuPont) were dispersed in 1 mL of water–ethanol solution at volume ratio of 2:1 by ultrasonicating for 1 h to form a homogeneous suspension. Then 5 μL of the mixture was loaded onto the glassy carbon electrode. Compensation for iR drop was used for all CVs. All potentials were reported versus the RHE based on the equation: *E*_RHE_ = *E*_Ag/AgCl_ + (0.197 + 0.0591 × pH). Current–potential data for Tafel plots were acquired by implementing controlled potential electrolysis in 15 mL of 0.1 m KOH solution at a variety of applied potentials in two‐compartment cells. The stable currents were measured at applied potentials ranging from 1.42 to 1.62 V in every 20 mV step for 600 s CPE experiments with the solution being gently stirred. CPE was recorded at 1.62 V in a fritted cell with ITO working electrode (5 × 5 mm^2^) with the catalyst (18.0 μL of the aforesaid suspension). The EIS was recorded on an ITO electrode over a frequency range from 0.1 Hz to 1 mHz at the amplitude of the sinusoidal voltage of 5 mV under different potentials ranging from 1.52 to 1.67 V. The TOF value was calculated by assuming that every nickel atom was involved in the catalysis (lower limit): TOF = *i*/(4*F* × *n*). Here, *i* (A) is the measured current, the number 4 means four electrons are required for the generation of one oxygen molecule, *F* is Faraday's constant (96 485.3 C mol^−1^), and *n* is the moles of the nickel atom on the electrode based on the amount of coated catalyst. Analysis of O_2_ produced in CPE experiments was conducted by using a calibrated Ocean Optics FOXY probe (Model NeoFox).

## Supporting information

As a service to our authors and readers, this journal provides supporting information supplied by the authors. Such materials are peer reviewed and may be re‐organized for online delivery, but are not copy‐edited or typeset. Technical support issues arising from supporting information (other than missing files) should be addressed to the authors.

Supplementary

Supplementary
