# Supplementary material for: Porous Nickel–Iron Oxide as a Highly Efficient Electrocatalyst for Oxygen Evolution Reaction
Source: Adv Sci (Weinh). 2015 Sep 10;2(10):1500199. doi: 10.1002/advs.201500199 (PMC5049451; doi:10.1002/advs.201500199)
Supplement: Supplementary file 1 — Supplementary [file ADVS-2-0n-s002.pdf]

Copyright WILEY-VCH Verlag GmbH & Co. KGaA, 69469 Weinheim, Germany, 2015.

ADVANCED SCIENCE
Open Access

Supporting Information

for *Adv. Sci.*, DOI: 10.1002/advs.201500199

Porous Nickel–Iron Oxide as a Highly Efficient Electrocatalyst for Oxygen Evolution Reaction

Jing Qi, Wei Zhang, Ruijuan Xiang, Kaiqiang Liu, Hong-Yan Wang, Mingxing Chen, Yongzhen Han, and Rui Cao**

Supporting Information

Porous Nickel-Iron Oxide as a Highly Efficient Electrocatalyst for Oxygen Evolution Reaction

Jing Qi,[a] Wei Zhang,*[,a] Ruijuan Xiang,[a] Kaiqiang Liu,[a] Hong-Yan Wang,[a] Mingxing Chen,[b] Yongzhen Han,[b] and Rui Cao*[,a,b]

[a] School of Chemistry and Chemical Engineering, Shaanxi Normal University, Xi'an 710119, China.

[b] Department of Chemistry, Renmin University of China, Beijing 100872, China.

*Correspondence and requests for materials should be addressed to W. Z. and R. C. (zw@snnu.edu.cn, ruicao@ruc.edu.cn)

General materials

All chemicals, including $Fe(NO_3)_3·9H_2O$ (99.999%, Alfa), $Ni(NO_3)_2·6H_2O$ (99.999%, Alfa), Tween 85 (Polyoxyethylenesorbitan Trioleate, Sigma-Aldrich), NaOH (98%, Sinopharm Chemicals), KOH (98%, Sinopharm Chemicals), and Nafion (5 wt%, DuPont), Ir/C (20 wt% of Ir, Premetek Co.) were purchased from commercial suppliers and used without further purification. Milli-Q water of 18 MΩ·cm was used in all experiments.

Synthesis of electrocatalysts

The materials were obtained through a co-precipitation strategy with the presence of Tween 85, a polysorbate surfactant with a boiling point above 100 °C. The oily polysorbate of 10 mL was firstly dissolved in 50 mL of 1 M NaOH solution at 60 °C under vigorous stirring, then a mixed aqueous solution containing $Ni(NO_3)_2$ and $Fe(NO_3)_3$ at different ratios (the total metal concentration is 2.5 M, volume is 20 mL) was added into the hot basic solution dropwisely. The suspension was stirred under the given temperature for 4 h. The solids were collected and washed by centrifugation in a sequence of water (2 times), acetone (1 time) and water (2 times) thoroughly. The obtained solids were dried in an oven at 60 °C and were subjected to further thermal treatment under different temperatures as required. The yield based on Fe is 90%. The bulk samples for comparison were synthesized from the same procedure except the addition of Tween. Samples are denoted as **Ni-X-Y**, in which **X** stands for the starting Ni percentage and **Y** stands for the treatment temperature in

degrees Celsius.

Characterization

Powder X-ray diffraction (XRD) patterns of the solids were recorded on a X-ray diffractometer (Rigaku D/Max2550VB+/PC, Cu Kα, λ = 1.5406 Å, 40 kV and 100 mA). Infrared spectra (IR) were recorded on an IR spectrometer (Bruker, Tensor27) using a standard KBr pellet technique. Thermogravimetric analysis was carried out by heating the dry powder sample at a rate of 5 °C/min with nitrogen flow at 100 mL/min over 25 °C to 800 °C in a TA Instruments SDT Q600. The carbon amount of the sample discussed in the main text was analyzed on a CHNS elemental analyzer (Elementar, Vario EL III). The morphologies of the solids were observed with transmission electron microscopy (TEM, FEI, Tecnai G2 F20) using an accelerating voltage of 200 kV. Energy-dispersive X-ray analysis (EDX) was conducted on an AMETEK Materials Analysis EDX equipped on the TEM. Scanning electron microscopy (SEM, FEI, Quanta 200) was referred for the observation of the electrocatalyst morphology on ITO electrodes. The high voltage was kept at 20 kV and the base pressure in the analyzer chamber was about 5×10^{-3} Pa. The Brunauer-Emmett-Teller (BET) surface areas and pore size distributions were measured in Micromeritics ASAP 2020 using the liquid nitrogen adsorption method. The X-ray photoelectron spectroscopy analysis of the samples was carried out on a Kratos AXIS ULTRA XPS. Monochromatic Al Kα X-ray (hν = 1486.6 eV) was employed for analysis with photoelectron take-off angle of 90° with respect to surface plane.

Correction of the binding energy was carried out using C 1s peak at 284.6 eV arising from the adventitious hydrocarbon.

Electrochemical studies

All electrochemical experiments were carried out using a CH Instruments (CHI 660E Electrochemical Analyzer) at 20 °C. Cyclic voltammograms (CVs) were obtained in 15 mL of 0.1 M KOH aqueous solution using a conventional three-electrode configuration with a 0.07 cm^2 glassy carbon (GC) electrode as the working electrode, saturated Ag/AgCl as the reference electrode, and Pt wire as the auxiliary electrode. The GC electrode was polished with α-Al_2O_3 of decreasing sizes (1.0 μm to 50 nm) and ultrasonically washed with deionized water and absolute ethanol before the loading of catalyst. The Pt wire auxiliary electrode was routinely ultrasonicated in 3 M nitric acid to remove any deposited pollutants. The working electrode was prepared through a drop casting method. Typically, 2 mg of sample and 30 μL of Nafion solution (5 wt%, DuPont) were dispersed in 1 mL of water-ethanol solution at volume ratio of 2:1 by ultrasonicating for 1 h to form a homogeneous suspension. Then 5 μL of the mixture was loaded onto GC electrodes. Compensation for iR drop was used for all CVs. All potentials were reported versus the reversible hydrogen electrode (RHE) based on the equation: $E_{RHE} = E_{Ag/AgCl} + (0.197 + 0.0591 \times pH)$. Current-potential data for Tafel plots were acquired by implementing controlled potential electrolysis in 15 mL of 0.1 M KOH solution at a variety of applied potentials in two-compartment cells. One compartment contained a Pt auxiliary

electrode and the other compartment contained the working electrode (ITO) and Ag/AgCl reference electrode. The stable currents were measured at applied potentials ranging from 1.42 to 1.62 V in every 20 mV step for 600 s CPE experiments with the solution being gently stirred. Controlled potential electrolysis (CPE) was recorded at 1.62 V in a fritted cell with ITO working electrode (5×5 mm^2) with the catalyst (18.0 μL of the aforesaid suspension). The electrochemical impedance spectroscopy (EIS) was recorded on an ITO electrode over a frequency range from 0.1 Hz to 1 mHz at the amplitude of the sinusoidal voltage of 5 mV under different potentials ranging from 1.52 V to 1.67 V. The TOF value was calculated by assuming that every nickel atom was involved in the catalysis (lower limit): TOF = i/(4F×n). Here, i (A) is the measured current, the number 4 means 4 electrons are required for the generation of one oxygen molecule, F is Faraday's constant (96485.3 C/mol), and n is the moles of the nickel atom on the electrode based on the amount of coated catalyst. Analysis of O_2 produced in CPE experiments was conducted by using a calibrated Ocean Optics FOXY probe (Model NeoFox). The experiment was performed in a three-compartment (Pt auxiliary electrode, working electrode, Ag/AgCl reference electrode) gas-tight electrochemical cell with a Schlenk connection on each compartment. Nitrogen gas was bubbled into a 0.1 M KOH solution (pH ~13.0) for 1 h under vigorous stirring to remove O_2. The phase shift of the O_2 sensor on the FOXY probe, recorded at 10 s intervals, was converted into the concentration of O_2 in the headspace using a two-point calibration curve (air, 20.9% O_2; and high purity N_2, 0% O_2). After recording the concentration of O_2 for 1 h in the absence of an applied

potential, CPE was performed at a potential of 1.62 V. O_2 signal was recorded for an additional 2 h after terminating the electrolysis.

Table S1. Physical information of the as-prepared electrocatalysts.

Sample ID	Ni-Fe added	Thermal treatment	Ni-Fe found[a]	Structure	BET surf. area (m^2/g)	Pore size (nm)
1	50:50	200 °C	5.5:94.5	Fe_2O_3	112	-
2	70:30	200 °C	15.1:84.9	$NiO/NiFe_2O_4$	84	-
3	80:20	200 °C	59:41	$NiO/NiFe_2O_4$	75	-
4	85:15	200 °C	63.7:36.3	NiO[c]	164	2.4
5	90:10	200 °C	76.3:26.7	NiO	156	-
6	98:2	200 °C	88:12	β–$Ni(OH)_2$	182	-
7	100:0	200 °C	99.9:0.1	β–$Ni(OH)_2$	176	-
8	85:15	RT	63.6:36.4	α–$Ni(OH)_2$	-	-
9	85:15	100 °C	60.7:39.3	α–$Ni(OH)_2$	-	-
10	85:15	300 °C	61.6:38.4	$NiO/NiFe_2O_4$	121	3.8
11	85:15	400 °C	62.2:37.8	$NiO/NiFe_2O_4$	60.5	6.3
12	85:15	500 °C	60.3:39.7	$NiO/NiFe_2O_4$	50.3	-
13[b]	85:15	200 °C	64.1:35.9	α–$Ni(OH)_2$	107	3

[a] Determined by XPS; [b] Sample without Tween;
[c] With around 3.1 mol% of Ni in the form of $NiFe_2O_4$.

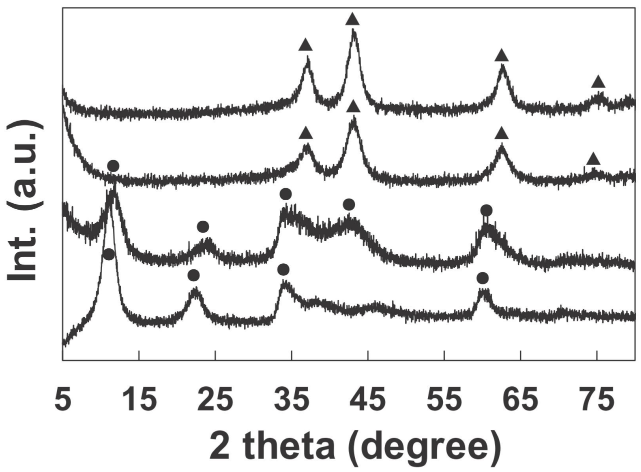

Figure S1. XRD patterns of **Ni-85-Y** samples without the addition of Tween during synthesis (**Y** from bottom to top: rt, 200, 250, 300). ●: α-$Ni(OH)_2$, ▲: NiO.

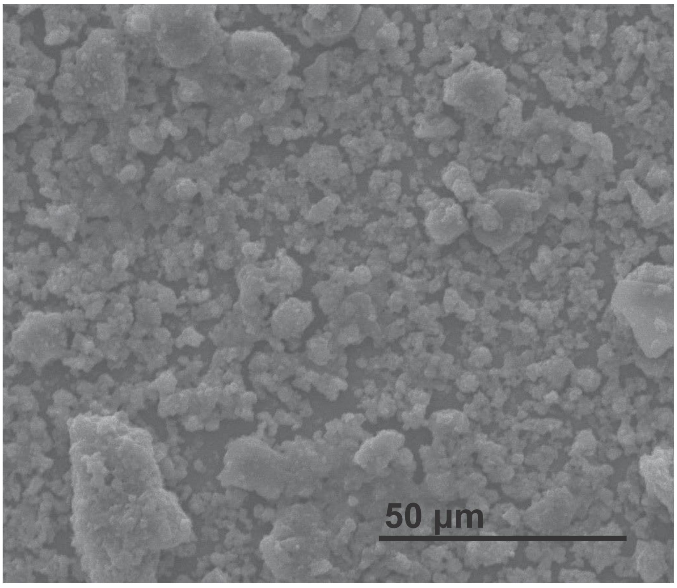

Figure S2. SEM image of the **Ni-85-200** electrocatalyst deposited on ITO electrode.

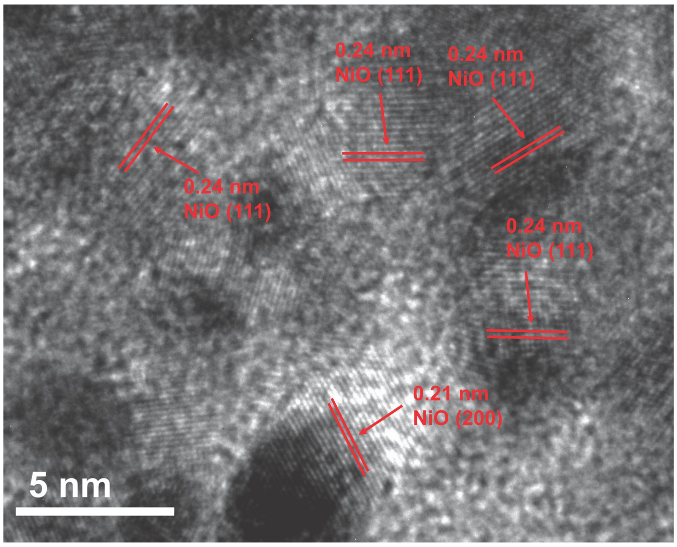

Figure S3. HRTEM image of the **Ni-85-200** electrocatalyst.

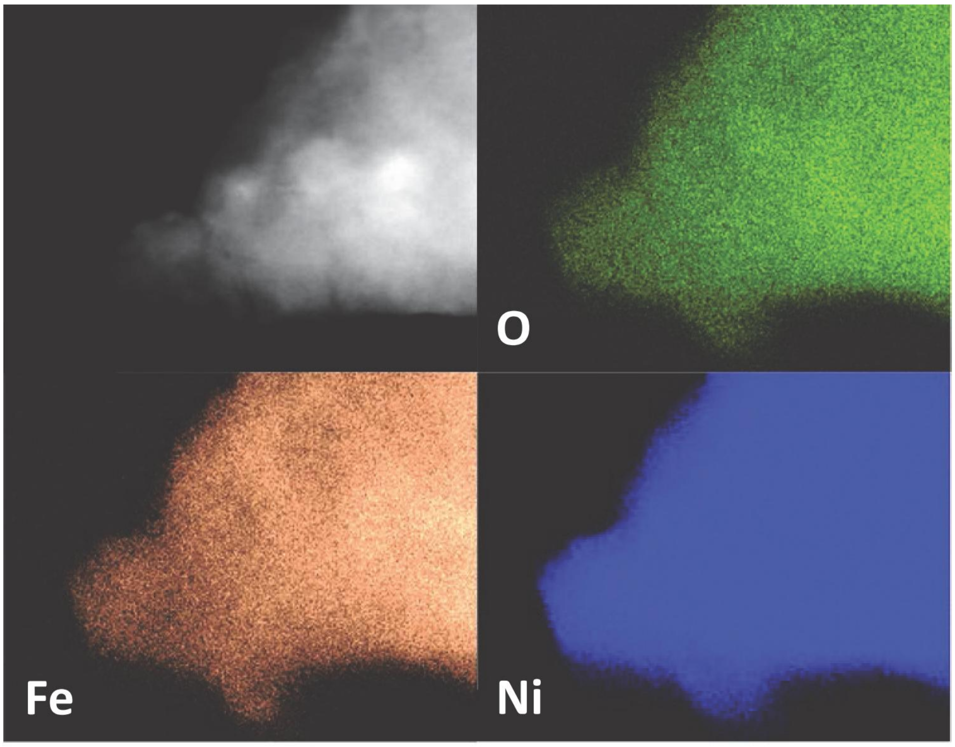

Figure S4. High angle annular dark field (HAADF) scanning transmission electron microscopy (STEM) image (top left) of the **Ni-85-200** sample, and its oxygen, iron and nickel energy-dispersive X-ray analysis (EDX) elemental mapping images.

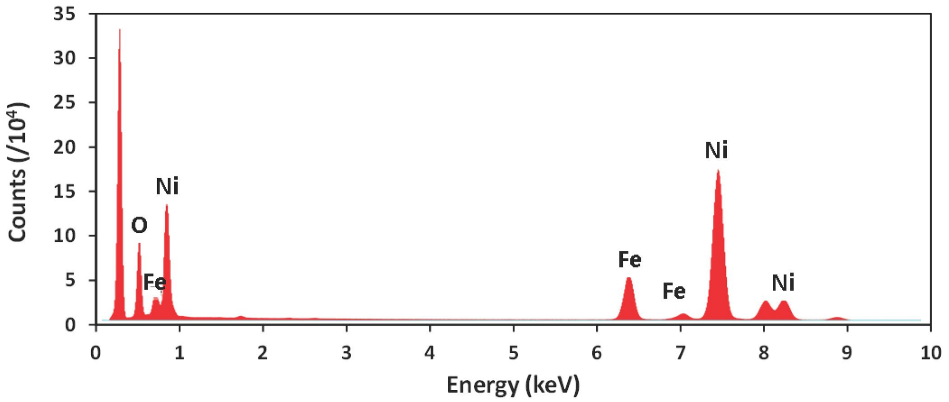

Figure S5. EDX analysis of the sample studied in Figure S4.

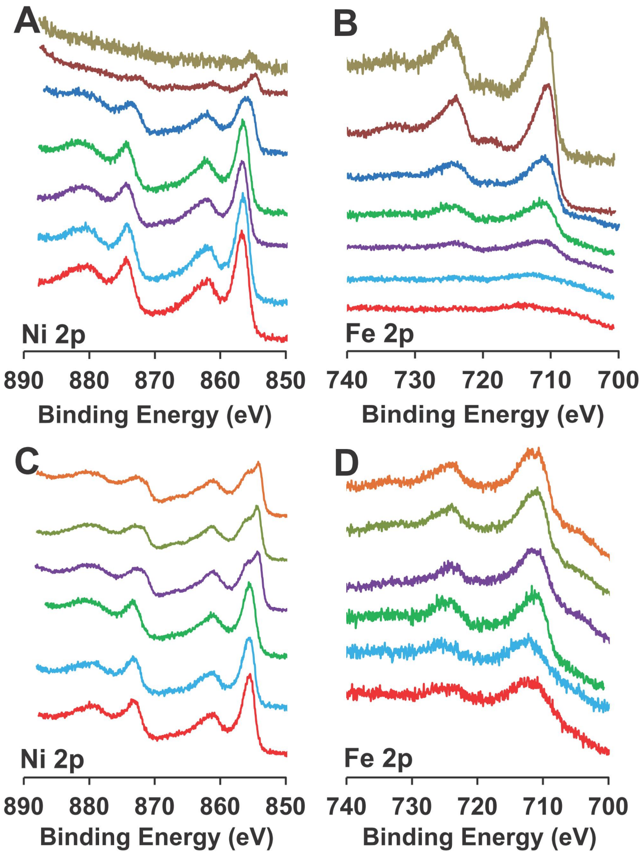

Figure S6. XPS spectra of Ni 2p and Fe 2p from the **Ni-X-Y** samples with different **X** (A, B. **X** from bottom to top: 100, 98, 90, 85, 80; 70, 50), and with different **Y** (C, D. **Y** from bottom to top: rt, 100, 200, 300, 400, 500).

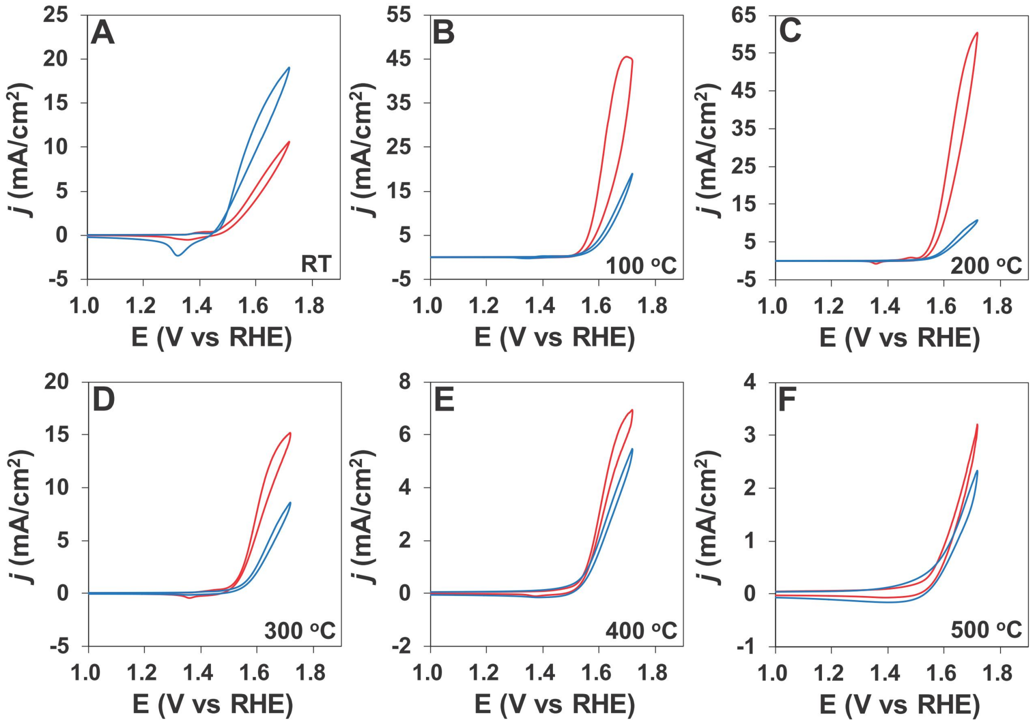

Figure S7. CVs of **Ni-85-Y** samples with (red line) or without (blue line) Tween.

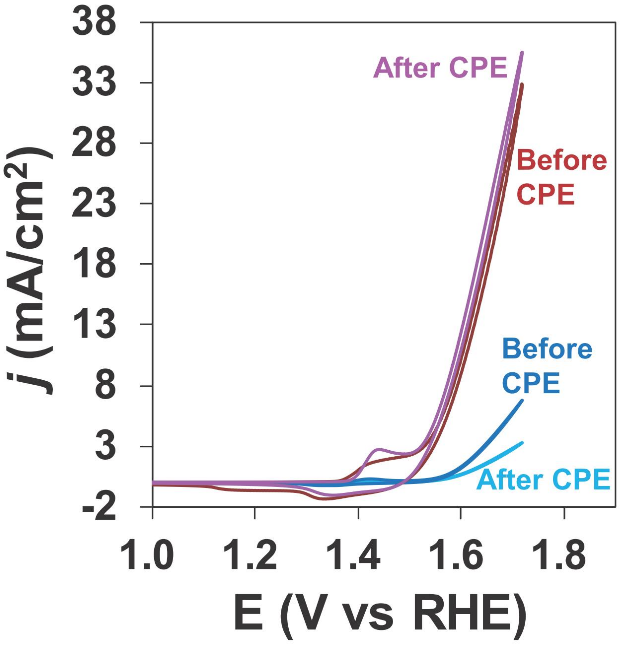

Figure S8. CVs on ITO electrodes before and after electrolysis from **Ni-85-200** (red lines) and **Ni-85-rt** (blue lines). Darker color lines are before electrolysis; lighter color lines are the CVs from the electrodes after electrolysis under 1.62 V in 0.1 M KOH for 12 h.

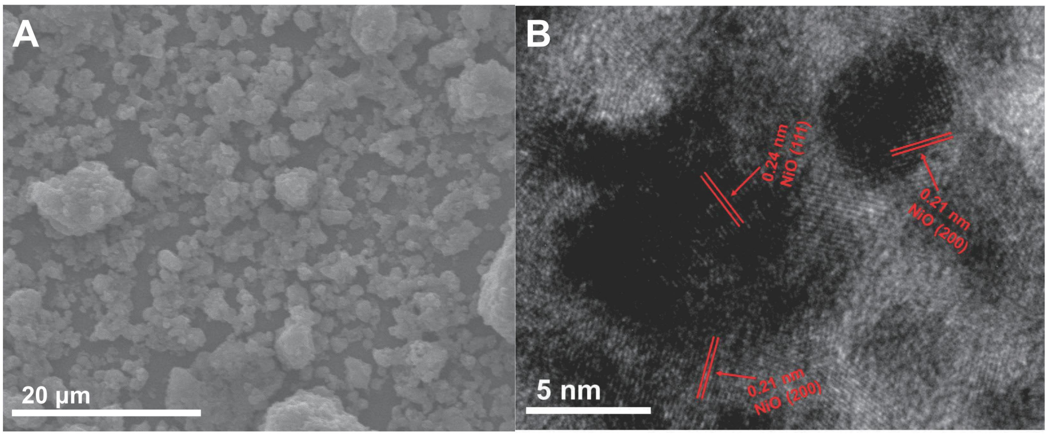

Figure S9. SEM (**A**) and HRTEM (**B**) images of the **Ni-85-200** samples after electrolysis under 1.62 V for 12 h in 0.1 M KOH.

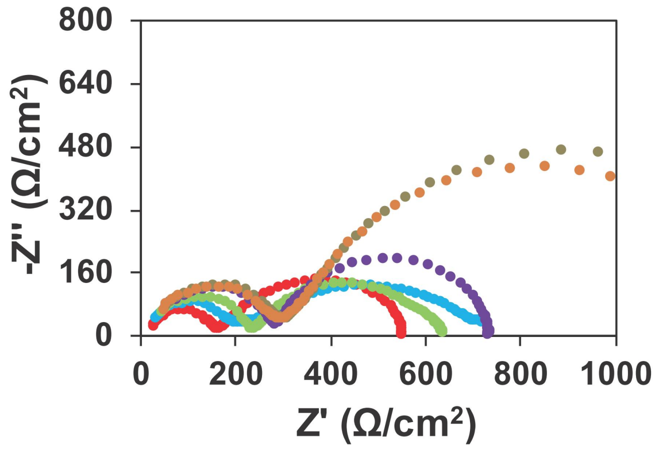

Figure S10. ESI Nyquist plots of the **Ni-85-Y** samples at 1.57 V. Red: **Ni-85-200**; Green: **Ni-85-100**; Azure: **Ni-85-rt**; Purple: **Ni-85-300**; Wheat: **Ni-85-400**; Gray: **Ni-85-500.**
